# Hospital Festivals and Meetings

**Published:** 1895-03-16

**Authors:** 


### HOSPITAL FESTIVALS AND MEETINGS.

### HOME HOSPITALS' ASSOCIATION.

The annual meeting of the supporters of the Home Hospitals' Association was held at 16, Fitzroy Square, on Tuesday, March 12th, Mr. Albert G. Sandeman in the chair. Mr. Almond Hind, the hon. secretary, commenting upon certain features of the report before the meeting (which was taken as read), said one more patient had been treated in 1894 than in the preceding year; the receipts were £84, and the expenses £130 more than in 1893. The chairman briefly reviewed the past history of the association, from its inception in 1877, when the number of beds provided was 14, to the present, when they had increased to 24. The association was formed for the benefit of patients who were able to pay for their accommodation and treatment, and to provide them with home nursing. At its commencement this institution was the first of its kind in London, now there were 40 similar institutions in and about London, all of which owed their existence in a measure to the successful example thus put before them. The Home Hospitals' Association's staff of nurses was excellent, and it consequently had the support of the most eminent members of the medical profession. It would be difficult to suggest any improvement in the management, but there was one thing which would add to the benefits now to be derived from the hospital, and that was a convalescent home attached to it. He thought it would be an excellent thing if the hospital's supporters would subscribe to this end. Sir Richard Quain seconded the adoption of the report, and drew attention to the special particulars which differentiated the institution from others of its kind, especially the facts that by its constitution it is not worked for profit and is self-supporting, and that patients can be treated by their own medical men. Nothing could have worked more successfully, and he heartily wished the institution God-speed. The re-election of the committee was then proceeded with, and *apropos* of votes of thanks to the medical and executive staff proposed by Dr. Hare, Mr. Hind said he felt a special debt of gratitude was due to Miss Pearson, the lady superintendent, and Miss Anstruther, her assistant, and the nurses for their work. The success of such an institution must depend largely upon the nursing staff, and he attached especial importance also to the permanence of the nurses as an item in this success. Dr. Leith Napier added a cordial tribute to the excellence of the nursing, and went on to say he wished it might be possible to reduce the fees in some cases, so as to embrace a class—clerks' wives, and others in similar positions, to whom a charge of four guineas a week was an insuperable hindrance to their participating in the benefits of the home. He thought this might be done by putting such cases into a large ward divided into cubicles—the plan to be carried out in another house. Mr. Sandeman said he fully sympathised with the suggestion, and Mr. Hind added that the committee had often had this question before their consideration, but he was afraid at present it was quite impossible to reduce the fees below three guineas, the lowest now charged, in certain cases, even that rate leaving them out of pocket. It was absolutely essential that the hospital should be self-supporting. The committee would be most happy to lower the charges whenever feasible. A vote of thanks to the chairman concluded the meeting.

### NEW HOSPITAL FOR WOMEN.

The annual meeting was held at the hospital on Monday, March 11th, the chair being taken, in the unavoidable absence of the Rev. Ll. Davies, by the Rev. Luke Paget. In regretting the loss of Mr. Davies' presence, Mr. Paget spoke warmly of the many valuable services he had rendered to the hospital for many years past. The report which was before the meeting was only what might have been expected, a record of work ever more and more widely appreciated and more and more entirely trusted. A special feature in the year's history was the journey undertaken in October last by Miss Ellerby, M.D., ophthalmic surgeon to the hospital, who had been requested to go to India to operate on the Maharanee of Jamnagar for cataract, an operation which had been entirely successful, the sight of both eyes being completely restored. Touching upon the influence of hospital work, Mr. Paget said he had recently come upon the St. Pancras Board of Guardians, and he had been much struck by the present tremendous levelling-up of medical requirements, and the feeling which existed with regard not only to the care of the sick, but of those suffering from the infirmities of advanced age in the workhouse itself. He thought, too, that there was now a great general growth of genuine medical interest in the public at large, a fact for which to be devoutly thankful. The adoption of the report was seconded by Mrs. Scharlieb, who expressed the great regret universally felt at the retirement of two old and valued members of the medical staff, Mrs. Marshall and Mrs. De La Cherois. There was pleasure in welcoming new friends, but it must always be a trying time for a hospital when changes in the *personnel* of its staff took place. There was one fact not mentioned in the report upon which a word might be said, and that was the existence of a small convalescent home in the shape of a cottage at Albury, near Tring, which acted as a most valuable supplement to the work of the hospital. Many a poor woman there regained health and strength after leaving the hospital before returning to her home. It was useless to restore a poor working woman to health if it were not to working health, and to send her back unfit to cope with the bad air and insufficient food of her normal surroundings. The hospital had lately received a great accession of strength to its consulting staff in the person of Dr. Cullingworth, one of the physicians of St. Thomas's Hospital, to whom they were much indebted for kindly consenting to give his valuable aid. Mrs. Garrett Anderson, in proposing the election of the Rev. Ll. Davies as vice-president, said he was one of the first supporters of the hospital, and without his courage and energy she did not think they would ever have had the courage to attempt such a thing as the raising of £50,000 for the building of the hospital. Mrs. Westlake said with regard to the cottage at Albury, that it ought to be understood that it was entirely supported by the efforts of Miss Bagster and Miss Walker, and not in any way by the hospital. The condition of the funds was very satisfactory, though the present balance was scarcely expected, and was due to a windfall in the shape of a sum resulting from a ball given in Lincoln's Inn Hall in December, by kind permission of the Benchers. In the future the enlargement of the hospital would have to be seriously considered. Additional accommodation for the nursing staff would be needed, and a pathological museum was also desirable. The meeting closed with a vote of thanks to the chairman, and tea and coffee was afterwards provided for the visitors, many of whom took the opportunity of going round the wards, which were looking especially pretty and cheerful, and were bright with flowers and trails of ivy, fresh from country lanes.

### LONDON TEMPERANCE HOSPITAL.

The annual meeting of the governors of this hospital was held in the hall of the hospital, when the report and balance-sheet of the year were adopted. During 1894 there were 1,044 in-patients, 5,155 out-patients and 5,965 casualties. The finances of the hospital are in a very unsatisfactory condition, and two of the wards, though furnished, cannot be opened. The total ordinary income for last year amounted to £5,096, while the total ordinary expenditure reached the sum of £9,134. The deficit, together with some expenses for repairs and building, was made by legacies and special donations, amounting to £2,930, and by a transfer from the endowment fund of £2,318. In the evening a public meeting of the friends and supporters of the hospital was held, when the chair was taken by Lord Battersea. The Rev. Canon Basil Wilberforce moved a resolution appealing for further support for the hospital, and thanking the president, the Duke of Westminster, the board of management, and the medical and nursing staff for their efforts on behalf of the institution. He said there was never a time when the claims of the hospital were so urgent as now, for the claims of the whole temperance movement were never so urgent as now. They were coming to a death-grapple with their great enemy. There was a peculiar value in an institution like this. Alcohol was not forbidden; it was used in absolutely necessary cases, like other drugs. He trusted that the result of the meeting would be a large increase in pecuniary support. Mr. T. H. Roberts, M.P., seconded, and the resolution was carried. Mr. Cash, one of the trustees, spoke strongly of the financial position of the hospital. They had that year drawn £2,000 from their endowment, and if that had to be done each year they had only two years to run, as they were resolved not to go into debt. Sir Benjamin W. Richardson said that last year they had 1,044 in-patients, and in not a single case had alcohol been administered. This had been given to only seventeen patients during their twenty-two years' existence. The results were quite as good as in any other institutions, and better in many respects. Other speakers followed.

### SAMARITAN FREE HOSPITAL.

The annual meeting of the governors of this hospital took place on Tuesday, March 5th. Very few governors were present, and the proceedings were purely formal. Dr. Savage was unable, from illness, to attend the meeting, and in his absence the chair was taken by Dr. C. H. Routh; the Rev. J. Huchons, Mr. Alban Doran, Dr. Bantock, Mr. Malcolm and Mr. Butler Smyth were also present. The report, which was duly adopted, was taken as read. It showed a satisfactory financial condition, the hospital being free from debt, though this result had, unfortunately, only been arrived at by selling out certain stock to meet particular claims; otherwise, during the past year, the income would have been equal to the expenditure. The report drew attention to the fact that a large number of the cases received into the hospital came from the country, and, therefore, though a London charity, the Samaritan Hospital must be said to have special claims upon the country for support. The hospital is free to the whole of England, being much used by patients from a distance. The percentage of cost of administration to maintenance is very low, and the hospital is said to stand first among those of its kind on account of the work done within its walls. The surgical work of the past year was reported to have had "most gratifying results." There had been no death from ovariotomy, and the mortality generally was very low. The accommodation for the out-patients was stated to be very inadequate, and the committee earnestly hoped that increased subscriptions might enable them to bring this department up to the desired standard of efficiency as soon as possible. In commenting upon the report, the Chairman thought the hospital was especially to be congratulated upon its freedom from debt. He spoke also of the wonderful results now obtained in certain operations which he remembered in years gone by would have been thought almost incredible, and of the revolution in surgery witnessed during the past fifty years. The Chairman, having touched very briefly upon the most salient features of the report, the meeting closed with the usual votes of thanks.

### LONDON HOMŒOPATHIC HOSPITAL.

The annual meeting was held in the board-room on March 7th, the Earl of Wemyss and March (president) in the chair. The secretary-superintendent, Mr. G. A. Cross read the report for the past year, the forty-fifth since the establishment of the hospital. Considerable progress was reported in the new building, which the committee hope will be ready for occupation in July next. Certain difficulties had arisen with neighbouring owners of houses as to assumed damage to "ancient lights," &c., and, in consequence, in order to avoid possible litigation and expense the original plans had been somewhat modified, unavoidably restricting "the area of the top floor of the building, and seriously affecting its upper architectural features." Full work had been maintained during the year, and the receipts from private nursing having notably decreased, the board were obliged to announce a deficit of £1,523 19s. 4d., the largest which they have yet had to face. The organisation of the Nursing Institute is to form a separate annexe of the hospital in future, in deference to the views of the medical staff, and the board especially wished to impress "upon the profession the propriety, and indeed necessity, of loyally availing themselves of the advantage of employing nurses retained by the hospital." Though the temporary hospital only contained forty beds, the number of in-patients treated during the year reached a total of 496, and the number of out-patients increased to 11,376 as against 10,832, the highest total of any previous year. By the kindness of the present Lord and Lady Ebury the hospital had become possessed of a life-sized portrait of the late Lord Ebury, its former president, which was originally presented to the late Lady Ebury by "many subscribers in recognition of his great services in the cause of homœopathy."

Lord Wemyss said he was sorry to see the receipts from private nursing had fallen off; he should hope, at any rate, at the present time all the nurses must be in full work. He was afraid this falling off must be in some measure due to the doctors not always when possible taking their nurses from this hospital. He was glad to be able to announce that the Duchess of Teck had written to ask the probable date of the opening of the new hospital, which might be taken as an earnest of her intention to be present.

The treasurer (Viscount Emlyn) made a brief financial statement, in the course of which he announced that Miss Durning Smith had just given 50 guineas and Lord Wemyss 100 guineas towards the funds of the hospital.

Miss Durning Smith, through whose exertions the increase of the building fund during the past year was mainly due, said she much regretted that a larger sum had not resulted, the amount raised in the last seven months being £3,000.

A few brief remarks were made by Dr. Yeldham, Dr. Dudgeon, Captain Cundy, and Dr. Knox Shaw. The latter, replying on behalf of the medical staff, commented upon a remark made by a former speaker with regard to the fads of the doctors which were being carried out in the new building. He was, himself, proud of being a faddist; he had been glad to see that the plans had been published, and very favourably criticised in The Hospital.

The report of the Convalescent Home at Eastbourne associated with the hospital was afterwards read. The financial state of this home is satisfactory, but is still without any endowment, and additional accommodation for men patients is sadly needed. The meeting closed with the usual votes of thanks.

